# The Relationship of Health Beliefs with Information Sources and HPV Vaccine Acceptance among Young Adults in Korea

**DOI:** 10.3390/ijerph15040673

**Published:** 2018-04-04

**Authors:** Jarim Kim

**Affiliations:** School of Communication, Kookmin University, Bugak Hall 603, 77 Jeongneung-ro, Seongbuk-gu, Seoul 136-702, Korea; jrkim@kookmin.ac.kr; Tel.: +82-2-910-5495

**Keywords:** HPV, immunization, vaccine, HBM, information source, communication, acceptability

## Abstract

Despite the HPV vaccine’s efficacy in preventing cervical cancer, its coverage rates among Asians are very low. To increase immunization coverage among these populations, understanding the psychological factors that affect HPV acceptability is critical. To this end, this study examined the relationships between multidimensional health beliefs and HPV vaccine acceptance, and what information sources effectively foster HPV vaccination-related health beliefs. Data were collected using a survey of 323 undergraduate students in Korea. Results showed that perceived susceptibility, perceived severity, perceived benefits, and perceived vaccine safety concerns predicted vaccine acceptance. Multiple dimensions of perceived barriers showed differing impacts on vaccine acceptance. In addition, interpersonal information sources were effective in boosting various health beliefs for HPV vaccination. The Internet also was effective in reducing social barriers, but the effects were opposite to those of social media. Theoretical and practical implications are discussed.

## 1. Introduction

In 2012, approximately 528,000 new cervical cancer cases were found, and 266,000 women lost their lives, ranking cervical cancer as women’s fourth most common cancer worldwide [1]. The human papillomavirus (HPV) vaccines (e.g., Gardasil^®^ and Cervarix^®^) have been found to reduce HPV-related diseases, including cervical cancer or genital warts, and are recommended for both males and females [2,3]. Despite the proven efficacy and safety of the vaccines, vaccine uptake rates have not been satisfactory due to lack of awareness and various other barriers, particularly in Asian countries [4].

Cervical cancer ranks second in causing morbidity and mortality for Asian women, implying that many of these deaths are preventable with the HPV vaccine [4]. However, the estimated HPV vaccination coverage rates in that region are very low. The full coverage rates among the females in their 10–20 years was 35.6% in North America, 31.1% in Europe, and 35.9% in Oceania, but remained 1.1% in Asia [5]. Prior research [6,7,8,9,10,11] has indicated that ethnicity and culture affect HPV vaccine acceptability, but little research has been conducted in Asia [12,13].

On the other hand, prior studies [14,15,16] reported that different information sources have differing impacts on vaccine-related psychological processes that further influence vaccine uptake. However, there is a lack of research on how various information sources influence the decision-making process for HPV vaccination with relative impacts.

To fill in these gaps, the current study examines (1) the psychological processes that affect decision making for HPV vaccination and (2) how the use of various information sources differently affects the health beliefs of Korean young adults, based on the health belief model (HBM) [17], one of the most utilized theories in health contexts [18].

### 1.1. Health Belief Model

A number of psychological factors, including beliefs, attitudes, and intentions, affect individuals’ decisions to engage in health behaviors [19,20]. Health behavior theories are useful in identifying critical precursors of health behaviors and guide behavior changes [21]. The HBM, in particular, has been found to be a useful tool for predicting vaccination behaviors [17,22,23]. HBM consists of four constructs, including perceived susceptibility (i.e., the belief that one is likely to get HPV or HPV-related diseases), perceived severity (i.e., the belief that HPV or HPV-related diseases are serious problems), perceived benefits (i.e., the perceived advantages of getting the HPV vaccine), and perceived barriers (i.e., the perceived impediments to engaging in the vaccination behavior) [17,24].

These health beliefs have been found to be associated with the acceptability of the HPV vaccine in general. Prior studies have revealed that perceived susceptibility, perceived severity, and perceived benefits are positively associated with the intention to get vaccinated against HPV [12,25,26], whereas various perceived barriers (e.g., concerns about vaccine safety, high vaccine cost) are negatively related with vaccinating intention [25,27,28].

Perceived barriers of getting the vaccine, in particular, has been treated as a one-dimensional variable mixed of various types of barriers such as vaccine safety, cost, or pain, but studies [23,29] have identified perceived barriers as being a multi-dimensional concept. This study treats perceived barriers as multidimensional since operationalizing the concept as one-dimensional may obscure a given barrier’s relative effects on different cultural groups. In addition to commonly referenced perceived barriers in the vaccination context (i.e., vaccine safety and logistic barriers) [12,23,30,31], prior research [8,9,10,11,32,33] has identified two barriers among Asians, which are social stigma about getting the HPV vaccine (e.g., being perceived as promiscuous or infidelity to their partners) and talking about sex-related topics (e.g., talking about issues dealing with sex topics is embarrassing and taboo). Based on these extant research findings, this study poses the following hypotheses (Hs), specifically extending the dimensions of perceived barriers.

**Hypotheses** **1** **(H1).** *Health beliefs have positive relationships with attitude.*

**Hypotheses** **1a** **(H1a).** *Perceived susceptibility has a positive relationship with attitude.*

**Hypotheses** **1b** **(H1b).** *Perceived severity has a positive relationship with attitude.*

**Hypotheses** **1c** **(H1c).** *Perceived benefit has a positive relationship with attitude.*

**Hypotheses** **1d** **(H1d).** *Perceived barrier (safety) has a negative relationship with attitude.*

**Hypotheses** **1e** **(H1e).** *Perceived barrier (logistics) has a negative relationship with attitude.*

**Hypotheses** **1f** **(H1f).** *Perceived barrier (talking) has a negative relationship with attitude.*

**Hypotheses** **1g** **(H1g).** *Perceived barrier (norms) has a negative relationship with attitude.*

**Hypotheses** **2** **(H2).** *Health beliefs have positive relationships with intention when the vaccine is free of charge.*

**Hypotheses** **2a** **(H2a).** *Perceived susceptibility has a positive relationship with intention (free).*

**Hypotheses** **2b** **(H2b).** *Perceived severity has a positive relationship with intention (free).*

**Hypotheses** **2c** **(H2c).** *Perceived benefit has a positive relationship with intention (free).*

**Hypotheses** **2d** **(H2d).** *Perceived barrier (safety) has a negative relationship with intention (free).*

**Hypotheses** **2e** **(H2e).** *Perceived barrier (logistics) has a negative relationship with intention (free).*

**Hypotheses** **2f** (**H2f).** *Perceived barrier (talking) has a negative relationship with intention (free).*

**Hypotheses** **2g** **(H2g).** *Perceived barrier (norms) has a negative relationship with intention (free).*

**Hypotheses** **3** **(H3).** *Health beliefs have positive relationships with intention when the vaccine is not free of charge.*

**Hypotheses** **3a** **(H3a).** *Perceived susceptibility has a positive relationship with intention (pay).*

**Hypotheses** **3b** **(H3b).** *Perceived severity has a positive relationship with intention (pay).*

**Hypotheses** **3c** **(H3c).** *Perceived benefit has a positive relationship with intention (pay).*

**Hypotheses** **3d** **(H3d).** *Perceived barrier (safety) has a negative relationship with intention (pay).*

**Hypotheses** **3e** **(H3e).** *Perceived barrier (logistics) has a negative relationship with intention (pay).*

**Hypotheses** **3f** **(H3f).** *Perceived barrier (talking) has a negative relationship with intention (pay).*

**Hypotheses** **3g** **(H3g).** *Perceived barrier (norms) has a negative relationship with intention (pay).*

### 1.2. Information Source Effects on Health Beliefs

Using different information sources regarding HPV and the vaccine has been found to have differential impacts on HPV vaccine uptake across various populations [14,16,34,35,36,37]. Vaccine uptake was positively related with relying on various vaccination information sources including healthcare providers [34,35,36,37], newspapers, brochures, family, and friends [34,36,37]. The relationships between Internet use and vaccination-related health beliefs, on the other hand, have not been consistent. A study [14] found that parents who used the Internet as a vaccination information source to gain information about getting their daughters vaccinated tended to perceive low benefits of getting the HPV vaccine, low susceptibility to HPV, and high barriers related to vaccine safety, while another study [16] revealed that parents who used the Internet for HPV vaccination information perceived greater susceptibility of their daughters to HPV and lower vaccine safety concerns, compared to parents who used other information sources. However, most studies have focused on actual vaccine uptake, limiting our understanding of the differing impacts various information sources have on respective health beliefs. Thus, the following research questions are posed.

**RQ1.***How do different information sources affect perceived susceptibility?*
**RQ2.***How do different information sources affect perceived severity?*
**RQ3.***How do different information sources affect perceived benefit?*
**RQ4.***How do different information sources affect perceived barrier (safety)?*
**RQ5.***How do different information sources affect perceived barrier (logistics)?*
**RQ6.***How do different information sources affect perceived barrier (talk)?*
**RQ7.***How do different information sources affect perceived barrier (norms)?*

## 2. Materials and Methods

### 2.1. Participants and Procedures

A cross-sectional study was conducted with those who had not received any HPV shots. Participants were recruited from undergraduate communication classes at one of the major universities in Seoul, Korea, using a traditional paper questionnaire. The purpose and basic information of the research were explained to the participants. Among 360 surveys distributed between November 2016 and March 2017, 37 incomplete responses were removed, leaving 323 valid responses remaining. Participation was voluntary and no rewards including extra credits were provided. Young adults (males under 22 and females under 27) are one of the major targets aimed at by the U.S. Department of Health and Human Services [38]. These age groups, due to HPV vaccine’s first introduction in 2006, had not been fully informed of this new vaccine when they were the prime target at the ages of 11–12 years. Thus, college students are an important target to be better understood. The institution to which the researcher belongs does not require such processes, but the researcher tried to comply with the ethical standard for conducting research and treating participants.

### 2.2. Measurements

Information sources for HPV and HPV vaccine were assessed by asking if participants have heard about HPV or the HPV vaccine from each of the sources including television, newspaper and magazines, radio, Internet (websites, blogs, forums, etc.), social media (Facebook, Twitter, Instagram, etc.), family and friends, and healthcare providers. Participants responded either “yes” or “no.” To measure key variables, three items with 7-point Likert scales ranging from 1 (strongly disagree) to 7 (strongly agree) were used and then averaged to form an index for respective construct, if not specified. Items for the health belief model were adapted from Kim and Nan [23] and Witte’s [39] Risk Behavior Diagnostic Scale. Three items were employed to measure perceived susceptibility to HPV (e.g., “It is likely that I will contract the HPV”), perceived severity of HPV (e.g., “I believe that the HPV will result in severe health problems”), and perceived benefits of the HPV vaccine (e.g., “I believe the HPV vaccine is effective in preventing the HPV”).

Items for perceived barriers to HPV vaccination, specifically, were adapted from prior studies [23,40]. Three items were used respectively to assess perceived vaccine safety-regarded barriers (e.g., “The HPV vaccine might cause short term problems, like fever or discomfort’’) and perceived logistical barriers (e.g., “It is hard for me to find a provider or clinic that is easy to get to”).

In addition, two more barrier constructs were developed based on prior research on Asians’ perceptions and attitudes toward the HPV vaccination, where cultural barriers were found to exist (e.g., premarital sexual behavior is not accepted) [7,11,41]. Three items assessed perceived barriers regarding talking to others about HPV or HPV vaccination (e.g., “Talking with friends regarding HPV or HPV vaccination makes me uncomfortable”) and another three items assessed perceived social norm-related barriers (e.g., “If I receive the HPV vaccine, other people will see me promiscuous”).

In assessing attitudes and intentions, items were adapted from a prior study [42]. Attitudes were measured by asking participants to rate on three 7-point scales (e.g., “very harmful–very beneficial”) as response to the statement “Getting vaccinated against the human papillomavirus (HPV) is…” Intentions when the vaccine was offered for free were measured by asking participants to respond using a 7-point Likert scale ranging from 1 (extremely unlikely) to 7 (extremely likely) to three statements (e.g., “How likely would you be to get the HPV vaccine sometime soon?”). Intentions when the vaccine cost 500,000 Korean won were measured using the responses to the same three statements.

### 2.3. Statistical Analysis

The data were analyzed using the SPSS Statistics 24 software (IBM Inc., Chicago, IL, USA). To test the hypotheses and research questions, a series of multiple regressions were utilized. In addition, to examine the structure of health beliefs, a confirmatory factor analysis (CFA) was conducted using AMOS Graphics (SPSS software). Age, gender, and annual income were included as control variables throughout the analyses, as these variables have been found to affect HPV vaccination-related beliefs, attitudes, and intentions [23,43,44,45]. Missing values were handled using the multiple imputation method.

## 3. Results

### 3.1. Descriptive Statistics

The mean age of participants was 21.30 (*SD* = 2.46), and 55.1% were female. About one-third (31.3%) of the sample reported an annual house income of 36–60 million won (one million won is approximately $900 US), and another one-third reported 60–120 million won (31.0%), followed by income levels of 12–36 million won (17.6%) and less than 12 million won (6.8%). Approximately six percent did not report their income levels. Table 1 presents the frequency of use of each information source. Table 2 presents the means, standardized deviation, and Cronbach’s alpha for the key variables.

### 3.2. Confirmatory Factor Analysis for Health Belief Constructs

To examine the multidimensional structure of health beliefs—especially those composed of perceived susceptibility, perceived severity, perceived benefits, and four dimensions of perceived barriers—a CFA with seven factors was conducted. The results demonstrated an acceptable model fit [46,47] showing χ^2^ (*df* = 168) = 434.049 (*p* < 0.001), the comparative fit index (CFI) = 0.930 and the root-mean square error of approximation (RMSEA) = 0.070 (90% C.I.; 062, 0.078).

### 3.3. Health Beliefs’ Influence on Attitudes and Intentions

The first set of hypotheses (H1a–H1g) tested the influence of health beliefs on attitude. As shown in Table 3, attitude was found to have a positive relationship with perceived benefits and a negative relationship with perceived barriers regarding vaccine safety, supporting H1c and H1d. Attitude, however, showed a positive relationship with perceived logistic barriers, which was the opposite outcome of that posed in H1e, and perceived susceptibility, perceived severity, perceived barriers to talking with others, and perceived social norms barriers were found to have no significant relationship with attitude. Thus, H1a, H1b, H1e, H1f, and H1g are rejected.

The second set of hypotheses (H2a–H2g) examined the influence of different health beliefs on intentions when the vaccine is offered for free. As shown in Table 3, intention (free shot) had positive relationships with perceived susceptibility, perceived severity, and perceived benefits and a negative relationship with perceived barriers regarding vaccine safety, supporting H2a–H2d. Intention (free shot) did not show any relationships with perceived logistic barriers, perceived barriers to talking with others, and perceived social norm barriers; thus, H2e–H2g are rejected.

The third set of hypotheses (H3a–H3g) tested health beliefs’ influence on intentions when the vaccine costs 500,000 Korean won. As Table 3 shows, intention (paid shot) showed positive relationships with perceived susceptibility, perceived severity, and perceived benefits and a negative relationship with vaccine safety-regarded barriers. Thus, H3a–H3d are supported. Intention (paid shot) showed no relationships with the other three barriers, rejecting H3e, H3f, and H3g. Additionally, male students showed greater intention to obtain the HPV vaccine than female students when it was offered for free.

### 3.4. Information Sources’ Influence on Health Beliefs

The research questions investigated the impacts of different information sources on health beliefs. Specifically, as Table 4 shows, hearing about HPV from health care providers increased perceived susceptibility to HPV (RQ1), while hearing about HPV from print media, including newspapers and magazines, increased perceived severity (RQ2). Hearing from different information sources did not influence the perceived benefits (RQ3), perceived vaccine safety barriers (RQ4), and perceived logistic barriers (RQ5). Using the Internet decreased perceived barriers to talking with others regarding the HPV vaccine (RQ6), and using the Internet along with family and friends as sources for HPV information decreased perceived barriers regarding social norms (RQ7). Hearing about the HPV on social media increased these perceived barriers (RQ6 and RQ7).

Additionally, sociodemographic characteristics predicted a number of health beliefs. Age was negatively associated with the perceived benefits of receiving the HPV vaccine, and income was positively associated with perceived vaccine safety concerns. Being male was positively associated with perceived susceptibility to HPV as well as perceived vaccine safety concerns, but it was negatively associated with perceived barriers regarding discussing the vaccine and social norms.

## 4. Discussion

This study aimed to explore how health beliefs affect vaccination-related attitudes and intentions as well as what information sources are effective in boosting health beliefs regarding HPV vaccination. First, as responses to the hypotheses, perceived susceptibility, perceived severity, perceived benefits, and perceived vaccine-safety concerns were found to predict attitudes and intentions to obtaining the HPV vaccine, as found in prior research [12,23,25,26].

One of the major contributions of this study is treating perceived barriers as multidimensional, especially incorporating possible cultural barriers. These research findings support prior research [12,23,30,31] that has identified various reasons for preventing these target populations from obtaining the HPV vaccine. As scholars [8,9,10,11,23,29,32,33] have argued, perceived barriers were composed of multiple dimensions. Vaccine-safety concerns were negatively associated with attitudes and intentions. Logistic barriers, interestingly, were found to have a positive relationship with attitudes, inconsistent with prior research [23]. One prior study revealed that people intending to receive the HPV vaccine expressed greater practical concerns [29]. Similarly, in this study, those who show interest in getting the vaccine along with a positive attitude may perceive more logistical barriers as they attempt to get the vaccine. Culturally relevant barriers—social stigma barriers and talking with others about the HPV vaccine—were not found to be associated with attitudes and intentions. It is fortunate to see these results, as it implies that the view of the HPV vaccine as socially unacceptable might not affect one’s own attitudes and intentions to get vaccinated. However, it may have indirect impacts on others’ vaccine acceptability and such perceptions may be easily amplified through the Internet, social media, and interpersonal communication, requiring future research.

The results for research questions generally supported prior studies finding associations between vaccine-supportive health beliefs and utilization of different information sources. In particular, interpersonal sources such as family and friends were found to be effective information sources, reducing social stigma barriers, and health care providers were found to be effective in increasing the perceived susceptibility to HPV. Recent studies [48,49] conducted in Italy revealed that HPV vaccine acceptance has a positive relationship with using health care providers as an information source, but a negative relationship with using friends as an information source. This inconsistency may stem from different outcome measures (i.e., HPV-related health beliefs versus vaccination status) or cultural differences in terms of information circulated in each respective society, which requires further research.

HBM posits that high susceptibility, severity, and benefits increase the likelihood of engaging in recommended behaviors while perceived barriers decrease such likelihood. Thus, the current findings support prior studies that showed positive relationships between the use of interpersonal sources and vaccine uptake [34,35,36,37]. Most traditional media, including television, radio, newspapers, and magazines, were not effective in affecting health beliefs for increased vaccine acceptance, except for print media’s association with perceived severity. It has been well-documented that reading news from newspapers, compared with watching television news, engages individuals in more cognitive information processing that allows attention to be paid to the details of provided information [50,51]. Print media, therefore, may have led participants to pay more attention to HPV-related severity information.

The Internet and social media showed opposite influences on perceived barriers. Both of these sources were not related with any other health beliefs showing inconsistent results from prior research [14,16]. The Internet was effective in decreasing barriers such as having HPV-related talks or social stigma, but social media increased such barriers. This suggests that content circulated on the Internet and social media differ: Internet content reduces unnecessary barriers to receive the HPV vaccine, while social media prevents people from getting the vaccine by increasing barriers. More generally, the Internet and social media have received considerable attention as health information sources [52,53], but the current findings imply that their functions need to be re-examined before being used as information sources for promoting health behaviors. Although it is not the focus of the current study, it is possible that the formats of social media (e.g., short texts, hyperlinks) may limit its ability to provide detailed information or may easily circulate rumors or misinformation (e.g., “promiscuous people get HPV”), requiring further research on the contents of respective information sources and their influences on health behaviors.

### 4.1. Limitations and Future Research Directions

The limitations of this study need to be acknowledged. First, the sample of this study was drawn from one college in Korea. To establish the generalizability of the study findings, studies with young adults in other regions in Korea and other Asian countries need to be conducted. Stratified sampling would be necessary in future research. Second, the cross-sectional nature of this study limits the causal relationships of information sources, attitudes, and intentions with HBM constructs. Experimental research is suggested to confirm such relationships. Third, although CFA confirmed multiple dimensions of perceived barriers, greater focus on scale development based on more comprehensive literature would benefit future research. Lastly, the use of different information sources was measured by self-report. It is possible that participants may have not correctly recalled their use of information source. Future studies are needed to employ other methods (e.g., media diary) [54,55] to better represent participants’ use of various information sources.

### 4.2. Practical Implications

This research also has several practical implications for promoting HPV vaccination. First, communication practitioners for HPV vaccine promotion need to strategically employ various information sources. This study suggests that mass media campaigns utilizing traditional mass media (e.g., television, radio, newspapers, and magazines) may not be of any effect. Promotions using interpersonal information sources are recommended. The Internet and social media are also suggested to be used to foster an atmosphere of social acceptance regarding the HPV vaccine. Especially misinformation or the stigmatizing atmosphere on social media needs to be monitored and managed so as not to negatively affect people’s vaccination acceptance. Communication practitioners also need to understand specific barriers that prevent a certain cultural group from getting the vaccine. This study found that social norm-related barriers (i.e., being viewed as promiscuous if they obtain the HPV vaccine) reduced young Koreans’ intention to get vaccinated even when the vaccine was offered for free. Different cultures have differing health-related beliefs, and understanding of such beliefs must precede further promotion of specific health behaviors.

## 5. Conclusions

The current study examined how various health beliefs influence HPV vaccine acceptance using HBM, especially highlighting the multidimensional nature of perceived barriers and what information sources effectively foster HPV vaccine-promoting beliefs. Results indicated that interpersonal sources are effective in increasing vaccine acceptance while traditional media generally are not. The Internet was found to remove social barriers, but social media increased such barriers. Perceived susceptibility, severity, and benefits positively predicted vaccine acceptance, but different perceived barriers showed differing impacts on vaccine acceptance.

## Figures and Tables

**Table 1:** Source of HPV Information (*n* = 323).

Source	*n*	(%)
Family and friends	140	(43.3)
Newspapers and magazines	96	(29.7)
Radio	10	(3.1)
Television	123	(38.1)
Internet	139	(43.0)
Social Media	97	(30.0)
Health Care Providers	61	(18.9)

**Table 2:** Means and Standardized Deviation of Key Variables (*n* = 323).

Variables	*Mean*	*SD*	*Reliability*
Perceived susceptibility	3.66	1.50	0.94
Perceived severity	4.69	1.16	0.87
Perceived benefits	5.25	1.07	0.86
Perceived barriers (vaccine safety)	4.15	0.88	0.61
Perceived barriers (logistics)	3.92	1.13	0.69
Perceived barriers (talking)	2.62	1.31	0.86
Perceived barriers (norms)	2.73	1.38	0.79
Attitude	5.33	0.93	0.93
Intention (free vaccine)	4.06	1.65	0.85
Intention (cost $390)	2.43	1.13	0.79

**Table 3:** Results of Multiple Regressions for Hypotheses.

Variable	Attitude	Intention (Free)	Intention (Paid)
Age	−0.029	−0.045	−0.003
Gender	−0.048	0.180 ***	0.110 *
Income	−0.004	−0.023	0.061
Perceived susceptibility	0.086 *	0.168 ***	0.168 ***
Perceived severity	0.095 *	0.139 ***	0.131 **
Perceived benefits	0.501 ****	0.281 ****	0.159 ***
Perceived barriers (safety)	−0.209 ****	−0.141 ***	−0.111 **
Perceived barriers (logistics)	0.120 ***	0.076	0.002
Perceived barriers (talking)	−0.036	−0.023	0.097
Perceived barriers (norms)	−0.058	−0.102 *	−0.096
Total R^2^ (adjusted)	0.411 ****	0.314 ****	0.136 ****

Notes: Entries are standardized beta coefficients; Gender: female = 1, male = 2. *p* < 0.10 *, *p* < 0.05 **, *p* < 0.01 ***, *p* < 0.001 ****.

**Table 4:** Results of Multiple Regressions for Research Questions.

Variable	Perceived Susceptibility	Perceived Severity	Perceived Benefits	Perceived Barriers (Safety)	Perceived Barriers (Logistics)	Perceived Barriers (Talking)	Perceived Barriers (Norms)
Age	−0.035	−0.147 **	−0.133 **	−0.004	−0.043	0.031	−0.001
Gender	0.398 ****	0.115 *	−0.109 *	0.152 **	0.094	−0.145 **	−0.188 ***
Income	0.034	0.013	−0.051	0.178 ***	0.021	0.040	−0.009
TV	−0.038	−0.033	−0.037	0.046	0.009	0.021	0.099
Newspapers & magazines	0.019	0.155 **	0.022	−0.089	−0.036	−0.004	0.037
Radio	0.037	−0.054	0.028	−0.066	0.071	0.061	0.085
Internet	−0.082	0.015	−0.107	0.045	0.007	−0.167 **	−0.161 **
Social media	0.013	−0.044	0.005	−0.029	−0.052	0.135 **	0.145 **
Family & friends	0.110 *	0.003	0.119 *	0.034	−0.116 *	−0.117 *	−0.246 ****
Health care providers	0.125 **	0.111 *	0.045	0.013	−0.095	−0.034	−0.051
Total R^2^ (adjusted)	0.206 ****	0.062 ***	0.006	0.032 **	0.035	0.054 ***	0.106 ****

Notes: Entries are standardized beta coefficients; Gender: female = 1, male = 2. *p* < 0.10 *, *p* < 0.05 **, *p* < 0.01 ***, *p* < 0.001 ****.
